# Synthesis and Plugging Performance of Nano-Micron Polymeric Gel Microsphere Plugging Agents for Oil-Based Drilling Fluids

**DOI:** 10.3390/gels9040290

**Published:** 2023-04-01

**Authors:** Kecheng Liu, Ren Wang, Kesheng Rong, Zebin Yin, Tiemei Lu, Yongsheng Yu, Yingying Li, Zexing Yang, Jie Yang, Zhen Zhao

**Affiliations:** 1 Engineering Technology Research Institute, PetroChina Xinjiang Oilfield Company, Karamay 834000, China; 2 CNPC Engineering Technology R&D Company Ltd., Beijing 102206, China; 3 National Engineering Research Center for Oil and Gas Drilling and Completion Technology, Beijing 102206, China

**Keywords:** oil-based drilling fluid, nano-micron, polymeric microsphere

## Abstract

As shale gas recovery progresses to deep layers, the wellbore instability during drilling in applications of oil-based drilling fluids (OBFs) becomes increasingly severe. This research developed a plugging agent of nano-micron polymeric microspheres based on inverse emulsion polymerization. Through the single-factor analysis with respect to the permeability plugging apparatus (PPA) fluid loss of drilling fluids, the optimal synthesis conditions of polymeric microspheres (AMN) were determined. Specifically, the optimal synthesis conditions are as follows: the monomer ratio of 2-acrylamido-2-methylpropanesulfonic acid (AMPS): Acrylamide (AM): N-vinylpyrrolidone (NVP) were 2:3:5; the total monomer concentration was 30%; the concentrations and HLB values of emulsifier (Span 80: Tween 60) were 10% and 5.1, respectively; the oil–water ratio of the reaction system was 1:1; the cross-linker concentration was 0.4%. The polymeric microsphere (AMN) produced via the optimal synthesis formula had the corresponding functional groups and good thermal stability. The size distribution of AMN ranged mainly from 0.5 to 10 μm. The introduction of AMND in OBFs can increase the viscosity and yield point of oil-based drilling fluids and slightly decrease the demulsification voltage but significantly reduce high temperature and high pressure (HTHP) fluid loss and permeability plugging apparatus (PPA) fluid loss. The OBFs with 3% polymeric microsphere dispersion (AMND) reduced the HTHP and PPA fluid loss by 42% and 50% at 130 °C, respectively. In addition, The AMND maintained good plugging performance at 180 °C. The AMN particles can block leakoff channels of artificial cores, effectively prevent the invasion of oil-based drilling fluids into formations and suppress pressure transfer. OBFs with 3% AMND enabled the corresponding equilibrium pressure to decrease by 69%, compared with that of the OBFs. The polymeric microspheres had a wide particle size distribution. Thus, they can well match leakage channels at various scales and form plugging layers via compression–deformation and packed accumulation, so as to prevent oil-based drilling fluid from invading formations and improve wellbore stability.

## 1. Introduction

As an important unconventional natural gas resource, shale gas attracts a lot of attention [1,2]. Due to the high clay mineral content of shale, the drilling process adopting water-based drilling fluids (WBFs) was found with severe shale hydration and swelling and frequent wellbore instability accidents, such as wellbore collapse, block caving and bit/string sticking, which hindered the smoothness of shale gas recovery [3,4,5,6]. Compared with WBFs, oil-based drilling fluids (OBFs) presented not only good lubrication and shale inhibition performances but also high thermal stability and easy control of rheological properties. Therefore, OBFs were extensively applied to shale gas drilling [7]. However, as shale gas recovery gradually shifted from shallow to deep layers, complex geology was more often encountered during drilling, and it was still challenging to deal with wellbore instability with OBFs [8]. The invasion of OBF filtrate could cause formation instability, and pressure transfer via micro-fractures may result in wellbore instability [9,10,11,12,13]. Given the abovementioned factors, one of the keys to improving wellbore stability is to strengthen the plugging performance of OBFs and reduce filtrate invasion into formations [14].

Conventional plugging materials, such as silica [15], ultrafine calcium carbonate [16], oxidized asphalt [17] and modified graphene [18], failed to deliver desirable plugging performance due to relatively uniform (narrow) particle size distribution, low deformability and inferior dispersion capacity. Nonetheless, the research and application of nano-organic plugging agents for OBFs had delivered some promising results. Xie et al. [19] synthesized hyperbranched polyamines with particle sizes of 3–350 nm, which were highly compatible with OBFs and could significantly reduce fluid loss at high temperature and pressure (HTHP). Moreover, this product at a concentration higher than 1% presented a plugging rate of 100% for artificial core samples with permeability of 10 × 10^−3^ μm. Geng et al. [20] developed surface-modified polystyrene microspheres using oleic acid-modified styrene. The average particle sizes of these microspheres in ethanol and water were 82 nm and 111 nm, respectively. With the increasing concentration of the polystyrene microspheres, the viscosity of OBFs grew, the demulsification voltage was stable, and the filtration loss dropped significantly. Du et al. [21] synthesized polystyrene microspheres with particle sizes of 200~1000 nm using styrene and lauryl methacrylate, which present high dispersion capacity and some oil absorption–expansion behavior. These microspheres successfully block filter membranes of 0.1 μm, 0.3 μm and 1 μm. Such nano-organic plugging agents were all detected to exhibit excellent plugging performance in laboratory tests. Their expanded particle size distribution improved the matching between plugging agents and leakoff channels. However, at present, research on plugging agents with particle sizes at the nano-micron scale is rarely reported.

Polymeric microspheres (PMs) with good deformation, dispersity, elasticity, and temperature resistance have been widely used to enhance oil recovery [22,23,24]. However, few studies have been conducted about PMs used as a plugging agent in OBDF. PMs with a size range from 1 μm to 5 μm were also synthesized, and they reduced the artificial core permeability to 18% [25]. Micron-sized PMs were synthesized through inverse emulsion polymerization [26,27]. These PMs had good performance when plugging discs with a pore diameter of 5~150 μm. Few studies were conducted using PMs with a micro-nano size as a plugging agent for BFs.

Based on the inverse emulsion polymerization, this research developed a nano-micron polymeric microsphere dispersion. Acrylamido-2-methylpropanesulfonic acid (AMPS), Acrylamide (AM) and N-vinylpyrrolidone (NVP) were used as monomers. The optimal synthesis conditions were identified via the single-factor analysis of the hydrophilic–lipophilic balance (HLB) value, oil–water ratio, monomer composition, total monomer concentration and cross-linker concentration with respect to the permeability plugging apparatus (PPA)-based filtration of drilling fluids. Moreover, the infrared spectrum, thermal stability, micro-morphology and particle size distribution of the polymeric microsphere were analyzed. The effects of different concentrations of the polymeric microsphere dispersion on the rheological and fluid loss properties of OBFs were studied, and the thermal tolerance of polymeric microspheres was investigated. The plugging performance for the sand disk with pore diameter of 3 μm was evaluated via the permeability plugging experiment, and the plugging capability for artificial cores was assessed via the pressure transfer test.

## 2. Results and Discussion

### 2.1. Optimization of Synthesis Conditions

#### 2.1.1. HLB

**Figure 1:**
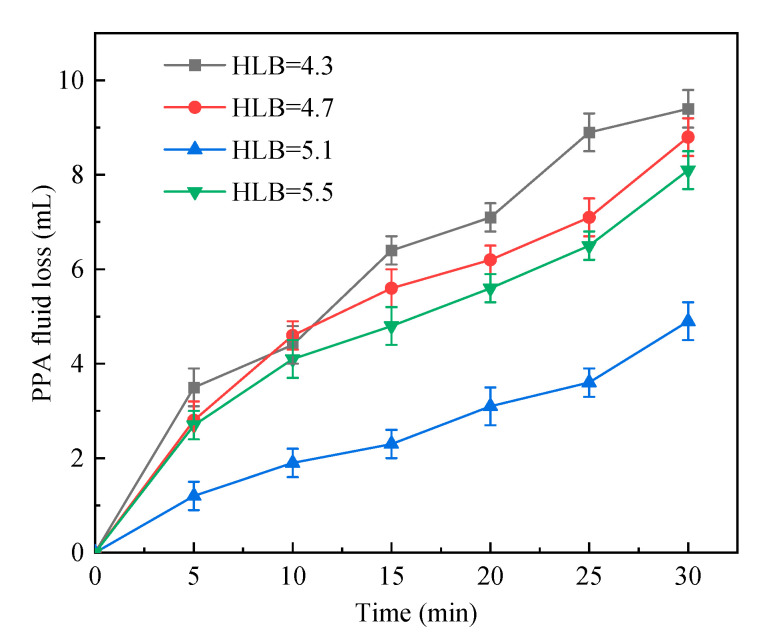
PPA fluid loss vs. time in cases of different HLB values.

The HLB value of emulsifiers has an important effect on their hydrophile and lipophilic performance. A reasonable HLB value ensures the reaction system is stable during the polymerization. Therefore, the HLB value of emulsifiers in the reaction system was changed from 4.3 to 5.5, and other conditions were fixed during the polymerization process, as shown in Table 1. Figure 1 shows that with the increasing HLB value, the PPA fluid loss first decreases and then increases. The PPA fluid loss was relatively high, with an HLB value of 4.3. In the presence of the emulsifier Tween 60, the HLB value grew, and the PPA fluid loss declined. As the HLB value reached 5.1, the PPA fluid loss dropped to the lowest, when the ratio of Span 80: Tween 60 was 9:1. However, with the further growth of the HLB value, the PPA fluid loss increased. When the HLB was 5.1, the system demonstrated best plugging performance, indicating that the optimal HLB value was 5.1.

#### 2.1.2. Oil–Water Ratio

**Figure 2:**
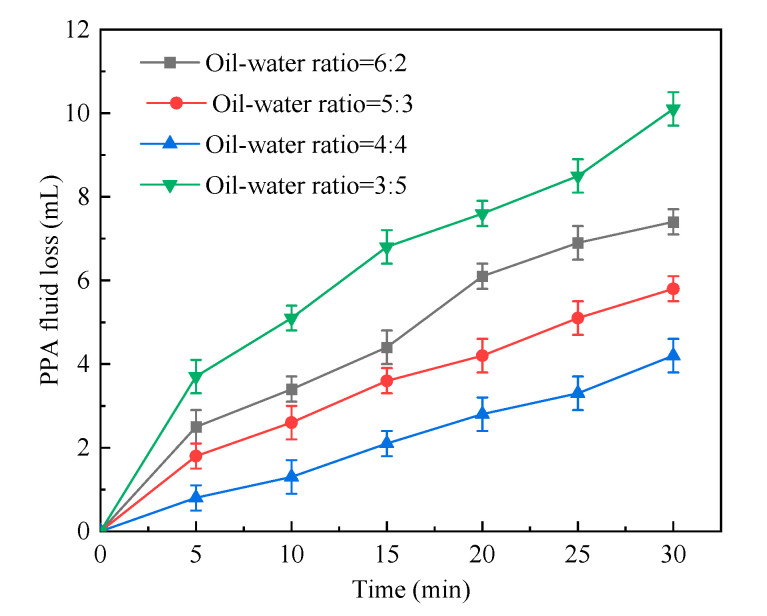
PPA fluid loss vs. time in cases of different oil–water ratios.

The oil phase was of benefit to absorb heat and prevent implosion during the reaction process. The suitable oil–water ratio promoted the polymerization of monomers. Therefore, the oil–water ratio of the reaction system was changed, while other synthesis condition remained unchanged as shown in Table 2. The test results were presented in Figure 2. With the decreasing oil–water ratio, the PPA fluid loss decreased first and then increased. The PPA fluid loss was rather high, with an oil–water ratio of 6:2. It decreased considerably as the oil–water ratio dropped to 5:3, and it reached the lowest values in the case of an oil–water ratio of 1:1. Subsequently, the oil–water ratio continued to decline. When the oil–water ratio was 3:5, the PPA fluid loss grew greatly. An excessively high oil–water ratio resulted in an excessively low solid content of the system, which led to inferior plugging performance and higher PPA fluid loss; with the reduction in the oil–water ratio, the solid content increased, and the plugging performance improved, which naturally lowered the PPA fluid loss. However, when the oil–water ratio was too low, too many monomers presented in the system stimulated the reaction and resulted in high odds of gelation. The continuous increase in solid content slowed down the heat dissipation of the system and even triggered coalescence. Under such circumstances, the plugging performance was low, and the PPA fluid loss grew. In the case of an oil–water ratio of 1:1, the weight and content of the solid phase were suitable, the emulsion of the system was stable and the plugging performance was relatively good. Therefore, the optimal oil–water ratio was considered to be 1:1.

#### 2.1.3. Monomer ratio

**Figure 3:**
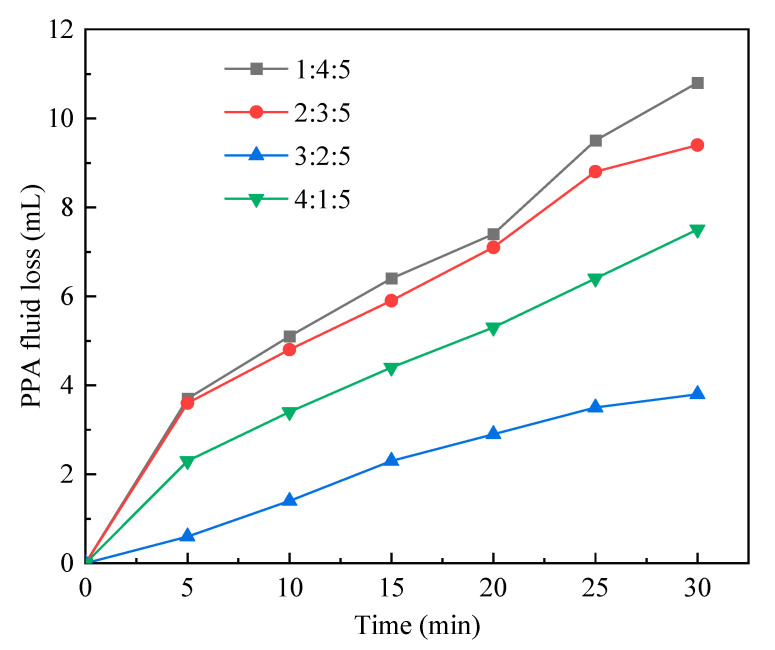
PPA fluid loss vs. time in cases of different monomer ratio (AMPS:AM:NVP).

The different monomer ratio could change the properties of polymeric microspheres. Therefore, the monomer ratio of the reaction system was changed while other reaction condition was fixed as shown in Table 3. Test results are shown in Figure 3. In the case of an increasing AMPS–AM ratio, the PPA fluid loss first declined and then increased. For the monomer ratio were 1:4:5 (AMPS:AM:NVP), the PPA fluid loss was relatively high. However, as the monomer ratio changed to 3:2:5, the PPA fluid loss grew; a considerable growth in the PPA fluid loss was associated with the 4:1:5. These findings were interpreted as follows: with the increasing content of AMPS, the hydration groups increased, which improved the plugging performance. However, as the AMPS content exceeded a threshold, the hydration effect was greater than the adhesion effect, and the plugging performance was therefore degraded. For the monomer ratio of 2:3:5, the polymeric microspheres were determined to display desirable adhesion and hydration effects associated with the best plugging performance. Hence, the optimal monomer ratio was 2:3:5.

#### 2.1.4. Total Monomer Concentration

**Figure 4:**
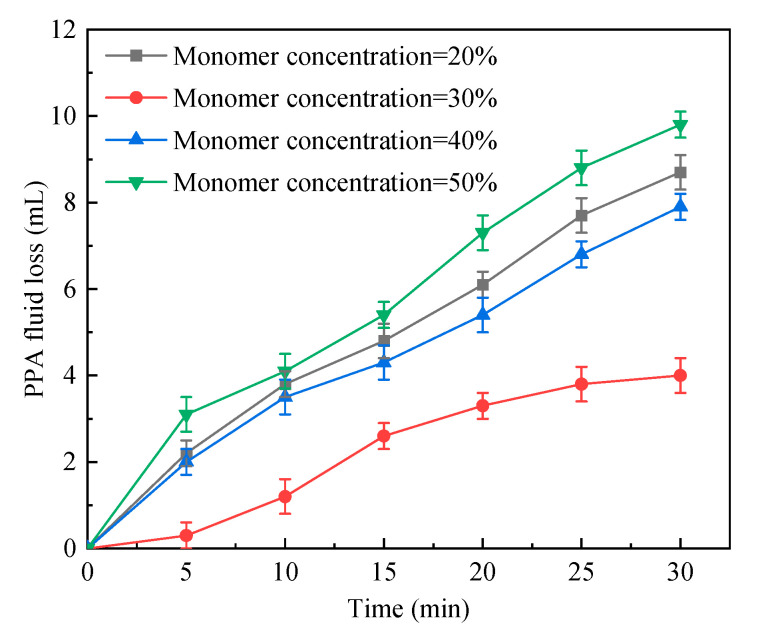
PPA fluid loss vs. time in cases of different total monomer concentrations.

The high polymeric microsphere content of dispersion was gained through improving total monomer concentration. The total monomer concentration of the reaction system was changed while other synthesis conditions remained unchanged as shown in Table 4, and the corresponding test results are shown in Figure 4. As the total monomer concentration grew, the PPA fluid loss first decreased and then increased. For a total monomer concentration of 20%, the PPA fluid loss was relatively high. It dropped greatly to the lowest value for a total monomer concentration of 30%. Nonetheless, as total monomer concentration continued to grow to 40%, the PPA fluid loss rate rose considerably. Such a growth trend of the PPA fluid loss remained as the total monomer concentration reached 50%. The reasons were that with the increasing total monomer concentration, the polymerization degrees of the synthesis product rose, which led to good plugging performance. However, an excessively high total monomer concentration caused the decline in the polymerization degrees, which reduced the plugging strength. When the total monomer concentration was too high, the heat dissipation was slow, the reaction accelerated, the steric hindrance effect for free radicals at the chain end intensified, and the pre-mature chain termination occurred. When the total monomer concentration was 30%, the polymerization degrees were the maximum, and the plugging strength was the strongest, associated with the lowest PPA fluid loss. Given such plugging performance, the optimal total monomer concentration was determined as 30%.

#### 2.1.5. Cross-Linker Concentration

**Figure 5:**
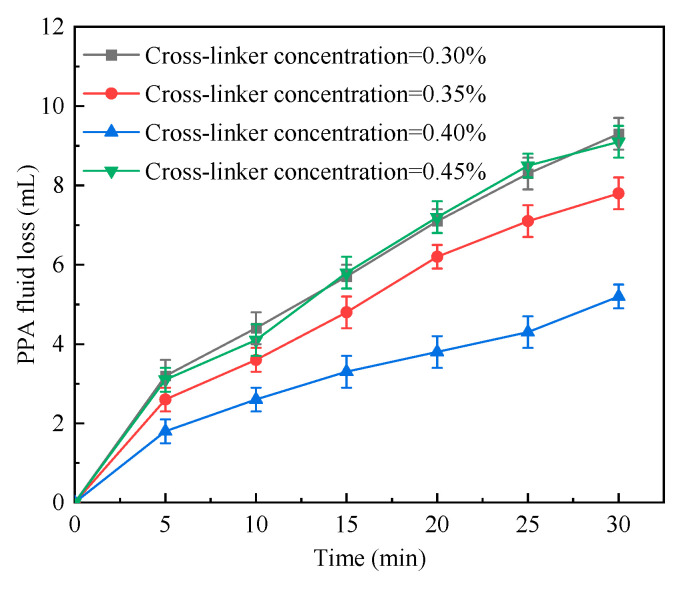
PPA fluid loss vs. time in cases of different cross-linker concentrations.

The addition of cross-linker concentration enabled the polymer to form a network structure during the synthesis process. This could enhance the polymer’s thermal stability. The cross-linker concentration was changed while other synthesis conditions were unchanged as shown in Table 5. The corresponding test results are illustrated in Figure 5. With the increasing cross-linker concentration, the PPA fluid loss first decreased and then increased. For a cross-linker concentration of 0.30%, the PPA fluid loss was relatively high; it dropped greatly as the cross-linker concentration increased to 0.35%; it was the lowest for a cross-linker concentration of 0.40%; however, as the cross-linker concentration further grew (to 0.45%), the PPA fluid loss started to increase. A rather low cross-linker concentration led to insufficient cross-linking of polymers and incomplete reaction. Consequently, the concentration of synthesized polymeric microspheres was low, the plugging performance was inferior, and the PPA fluid loss was high. Nevertheless, the growth of the cross-linker concentration promoted cross-linking and increased the strength of polymeric microspheres, which improved the plugging performance and reduced the PPA fluid loss. Yet, it should be noted that with the continued growth of the cross-linker concentration, the synthesized polymeric microspheres became weaker and more brittle due to excessive cross-linking. This resulted in the degradation of the plugging performance and the growth of PPA fluid loss. Clearly, for a cross-linker concentration of 0.40%, the polymers were well cross-linked, the reaction was rather sufficient, and the prepared polymeric microspheres had desirable strength and optimal plugging performance. Therefore, the optimal cross-linker concentration was 0.40%.

Finally, based on the above single-factor analysis, the optimal synthesis conditions of polymeric microspheres were as follows: the monomer ratio of AMPS:AM:NVP were 2:3:5; the total monomer concentration was 30%; the emulsifier concentration and HLB value of emulsifier (Span 80: Tween 60 = 9:1) were 10% and 5.1, respectively; the oil–water ratio was 1:1; the cross-linker concentration was 0.4%.

### 2.2. Material Characterization

#### 2.2.1. Infrared Spectroscopy

**Figure 6:**
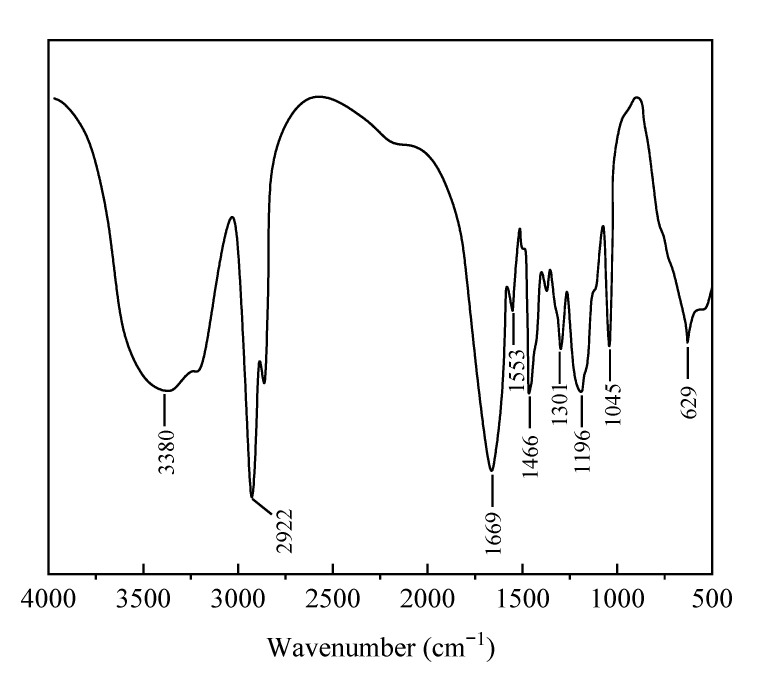
Infrared Spectrum of polymeric microspheres.

The infrared spectrum of AMN (Figure 6) showed that the characteristic absorption peak of symmetrical stretching vibration of −NH_2_ in AM was at 3380 cm^−1^; that of asymmetrical stretching vibration of amide groups in AM was at 1669 cm^−1^ [28]; for sulfonic acid groups in AMPS, the characteristic absorption peak of asymmetric stretching vibration was at 1196 cm^−1^, and that of symmetric stretching vibration of sulfonic acid groups was at 1045 cm^−1^; the absorption peak of C-S in AMPS was at 629 cm^−1^; the stretching vibration peak of −CH was at 2922 cm^−1^; the absorption peak of −CON in NVP was at 1466 cm^−1^; the absorption peak of −C=O in NVP was at 1301 cm^−1^. The characteristic peaks of AM, AMPS and NVP are included in the infrared spectrum, indicating that the three monomers had been successfully copolymerized.

#### 2.2.2. Thermal Stability Analysis

**Figure 7:**
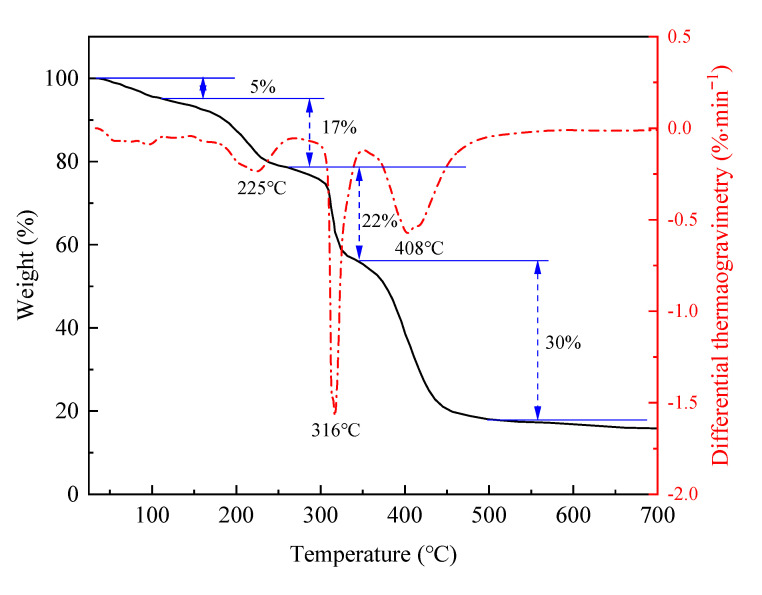
Thermogravimetric analysis of polymeric microspheres.

The thermogravimetric variation of AMN is shown in Figure 7. The thermal decomposition of AMN could be divided into four stages: 43 °C~105 °C, 105 °C~257 °C, 257 °C~342 °C and 342 °C~512 °C. The first stage highlighted the loss of free water, and the second stage was the loss of bound water. The third stage was characterized by the side chain breaking of polymeric microspheres, and the degradation rate was the highest at 316 ℃ [29]. The fourth stage featured the breaking of carbon chains of polymeric microspheres, and the fastest degradation rate occurred at 408 °C. The abovementioned results demonstrated that the AMN were stable at a temperature below 300 °C and thus had high thermal stability.

#### 2.2.3. Scanning Electron Microscopy and Particle Size Distribution Analysis

**Figure 8:**
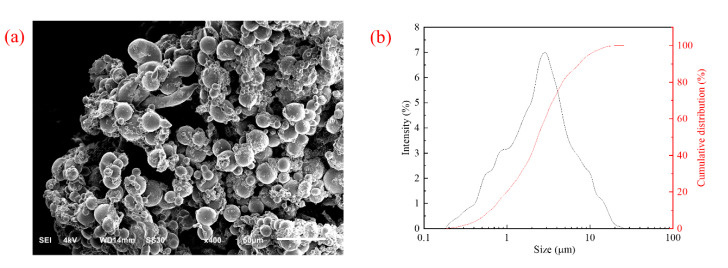
**(a)** SEM image and (**b**) particle size distribution.

The AMN appeared to be spheres with different diameters in Figure 8a. These spheres gathered together due to surface physical effects, and the particle sizes were between the micron and nano scales. As suggested by the frequency distribution of particle sizes, the particle size of polymeric microspheres was 0.15–25.00 μm (mostly 0.50–10.00 μm), suitable for plugging micron-scale pores and fractures in Figure 8b.

### 2.3. Plugging Performance

#### 2.3.1. Rheological and Filtration Properties

**Figure 9:**
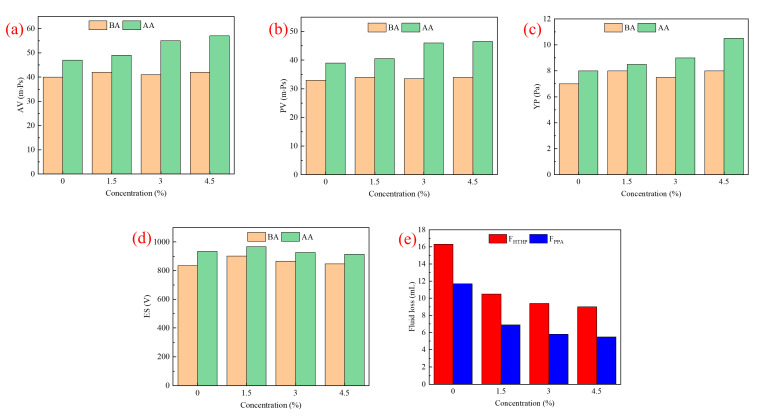
Values of the (**a**) AV, (**b**) PV, (**c**) YP, (**d**) ES and (**e**) F_HTHP_ and F_PPA_ of OBFs with different concentrations of AMND before and after aging (BA and AA) at 130 ℃.

The compatibility of AMND with OBFs was evaluated with respect to the changes in rheological, emulsifying and filtration properties of OBFs (1.6 g/cm^3^) with various concentrations of AMND before and after aging (BA and AA) for 16 h at 130 °C. The test results are shown in Figure 9. With increasing AMND concentration, except for the viscosity and yield point of the system that are found with considerable growths after hot rolling, other properties of the system were stable. Moreover, the HTHP fluid loss (FL_HTHP_) and PPA filtration (F_PPA_) of the system decreased significantly in the presence of AMN, which indicated that the AMN could effectively improve the filtration performance of the system. The test results suggested that the plugging agent was highly compatible with OBFs.

#### 2.3.2. Thermal Tolerance

**Figure 10:**
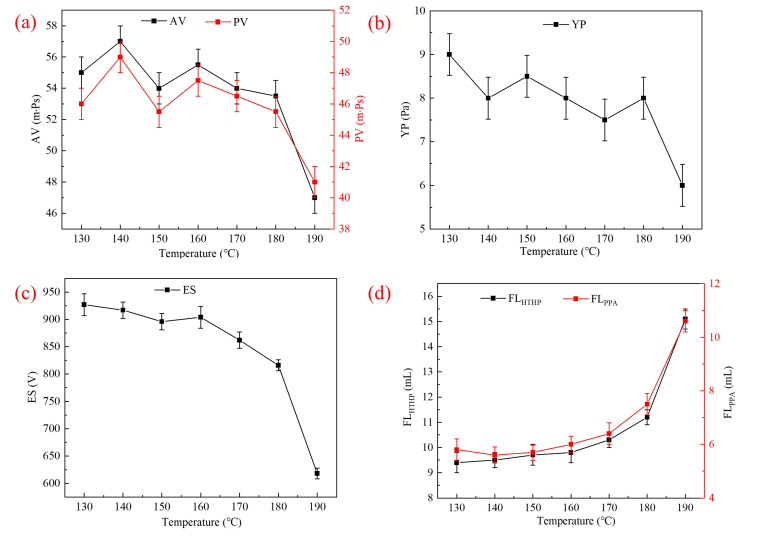
Values of (**a**) AV and PV, (**b**) YP, (**c**) ES and (**d**) F_HTHP_ and F_PPA_ after aging (AA) at different temperatures.

The thermal tolerance of AMN was evaluated by testing the performance of OBFs with 3% AMND under different temperature conditions. The test results are shown in Figure 10. As the hot rolling temperature increased from 130 °C to 180 °C, the yield point and demulsification voltage of drilling fluids declined—specifically, the yield point decreased from 9.0 Pa to 8.0 Pa, and the demulsification voltage decreased from 927 V to 816 V. Yet, the rheological and emulsifying properties remained stable. The HTHP fluid loss increased from 9.4 mL to 11.2 mL, and the PPA fluid loss increased from 5.8 mL to 7.5 mL, which were both far lower values than those of OBFs. However, for an elevated hot rolling temperature of 190 °C, the yield point and demulsification voltage decreased greatly—the demulsification voltage dropped below 800 V. Moreover, the fluid loss increased significantly to a value close to that of OBFs, indicating that the plugging performance was considerably degraded. The above analysis showed that the AMN had high thermal tolerance of up to 180 °C.

#### 2.3.3. Pressure Transfer

**Figure 11:**
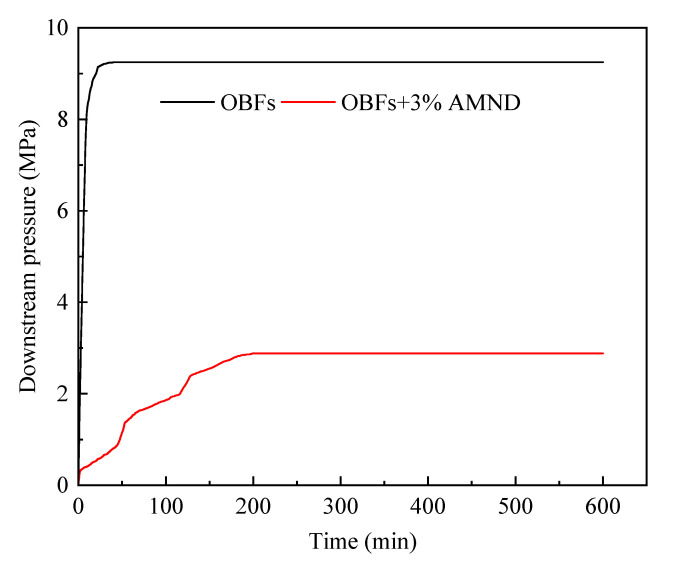
Experimental results of the pressure transfer test.

The pressure transfer curve is given in Figure 11. In the case of OBFs flowing in cores, the downstream pressure increased to the maximum within a short time. Nonetheless, it required two hours for the downstream pressure to peak, after adding 3% AMND into OBFs, and moreover, the corresponding equilibrium pressure decreased by 69% compared with that of the OBFs. It was suggested that the AMN effectively plugged the pores of cores and prevented the transfer of upstream pressure to the downstream, which indicated good plugging performance [22].

## 3. Conclusions

Polymeric microspheres (AMN) were synthesized via inverse emulsion polymerization using 2-acrylamido-2-methylpropanesulfonic acid (AMPS): Acrylamide (AM): N-vinylpyrrolidone (NVP) as polymerization monomers. The AMN was characterized by infrared spectroscopy, thermogravimetric analysis, scanning electron microscopy and dynamic light scattering instrument. The results showed that the AMN synthesized by the optimized formulation contained the corresponding functional groups and had good thermal stability. The particle size distribution of AMN ranged from 0.15 to10 μm. The rheological and plugging performance of oil-based fluids (OBFs) with polymeric microsphere dispersion (AMND) were investigated using the high temperature and pressure (HTHP) filtration test, permeability plugging apparatus (PPA), and pressure transfer test. The AMND was highly compatible with OBFs. With increasing AMN concentration, the viscosity and yield point of OBFs increased, but the demulsification voltage, HTHP and PPA fluid loss decreased. The oil-based fluid (OBs) with 3% AMND decreased the HTHP and PPA fluid loss by 42% and 50% at 130 °C, respectively. The 3% AMND still presented desirable plugging performance at 180 °C. The AMN could effectively plug pores and micro-fractures of artificial cores, prevent OBFs from invading cores, and lead to a downstream pressure far lower than the upstream pressure. The introduction of 3% AMND enabled the equilibrium pressure of OBFs to decrease by 69%. AMN with a wide size distribution could be well matched with multi-scale pores or cracks and form plugging layers after compression–deformation and packed accumulation so as to prevent OBFs from entering formations and improve wellbore stability.

## 4. Materials and Methods

### 4.1. Materials

Acrylamide (AM), 2-acrylamido-2-methylpropanesulfonic acid (AMPS), N-vinylpyrrolidone (NVP), N, N’-methylenebisacrylamide (MBA) and ammonium persulfate (APS) were provided by Beijing Mindrida Technology Co., Ltd., Beijing, China. Tween 60, sodium hydroxide and Span 80 were all purchased from Sinopharm Chemical Reagent Co., Ltd., Shanghai, China. Deionized water was prepared using the water purification equipment of our laboratory. In addition, 3# white oil was supplied by Jinan Luwenhao Chemical Co., Ltd., Jinan, China.

### 4.2. Synthesis of Polymeric Microspheres(AMN)

The nano-micron polymeric microspheres were synthesized as follows: first, a given amount of AMPS monomers was added into a beaker filled with a certain amount of deionized water and the pH value of the solution was adjusted to neutrality. A given amount of AM, NVP monomers and cross-linker MBA was successively added into the solution and stirred for 20 min. The mixture then was added to 3# white oil with a certain concentration Tween 60 and Span 80 and stirred for 30 min at 460 rotations per minute (RPM) under nitrogen. The initiator APS was added to the compound, and the reaction was kept for 12 h. The final product was polymeric microsphere dispersion (AMND). The polymeric microsphere dispersion was demulsified with ethanol and then the solid phase of AMN was gained via centrifuging. The product was dried at 65 °C to obtain solid microsphere powder (AMN).

### 4.3. Characterization Methods of AMN

The microsphere powder AMN was pressed into the test disc for infrared spectroscopy, after being mixed with potassium bromide. The used instrument was the Nicolet iS10 FT-IR spectrometer (manufactured by U.S.-based Thermo Nicolet Corporation, Waltham, MA, USA).

The thermal stability of AMN was tested using the TG 209 F3 Tarsus thermo-microbalance (a thermogravimetric analyzer manufactured by NETZSCH, Selb, Germany). In a nitrogen atmosphere, the crucible containing the sample was placed in the analyzer and heated from room temperature to 700 °C at the rate of 10 °C/min.

The microscopic morphology of AMN was measured via scanning electron microscopy (SEM). The AMN powder was gold-coated using a sputter coater.

The particle size distribution of AMN in ethanol was measured by a dynamic light scattering instrument 2000ZD (manufactured by Jinan Winner Particle Instrument Stock Co., Ltd., Jinan, China).

### 4.4. Plugging Performance Test

The formulation of base OBFs was 85% 3# white oil + 3% main emulsifier + 2% auxiliary emulsifier + 1% organoclay + 2% CaO + 15% brine (30% CaCl_2_) + 3% oxidized asphalt + barite. Polymeric microsphere dispersions with different concentrations were added into OBFs, and the mixture was aged for 16 h at the experimental temperature using the roller oven (manufactured by Qingdao Tongchun Oil Instrument Co., Ltd., Qingdao, China) after high-speed stirring. The high temperature and pressure (HTHP) filtration test was performed for aged samples using the 42-Model HTHP fluid loss meter (manufactured by Qingdao Haitongyuanda Special Instrument Co., Ltd., Qingdao, China). The rheological parameters and demulsification voltage (ES) of the OBFs before and after aging were measured at 65 °C using the ZNN-D6B six-speed viscometer, and the apparent viscosity (AV), plastic viscosity (PV) and yield point (YP) were calculated as per the relevant codes of the American Petroleum Institute (API).

(1) $$AV=\theta_{600}/2\;(mPa\cdot s),$$

(2) $$PV=\theta_{600}-\theta_{300}\;(mPa\cdot s),$$

(3) $$YP=0.48\times (\theta_{300}-PV)\;(Pa),$$

where the θ_600_ was the viscometer reading value of samples at 600 RPM, and the θ_300_ was the viscometer reading value of the sample at 300 RPM.

The HTD18984 permeability plugging apparatus (PPA manufactured by Qingdao Haitongda Special Instrument Co., Ltd., Qingdao, China) was used to evaluate the plugging performance of AMN, using a sand disc with a pore diameter of 3 μm. The plugging performance of artificial cores was assessed in accordance with the variation of downstream pressure with time during a pressure transfer test. The upstream pressure was set at 10 MPa; the pump rate was constant at 5 mL/min.

## Figures and Tables

**Table 1:** The synthesis conditions of AMN other than HLB.

Synthesis Conditions	Various Parameters
Oil–water ratio	1:1
Monomer ratio (AMPS:AM:NVP)	1:4:5
Total monomer concentration	30%
Cross-linker concentration	0.4%
Emulsifier concentration	10%
Initiator concentration	0.08%
Reaction temperature	50 °C
Reaction time	12 h
Rotation speed	460 rotations per minute (RPM)

**Table 2:** The synthesis conditions of AMN other than oil-water ratio.

Synthesis Conditions	Various Parameters
HLB	5.1
Monomer ratio (AMPS:AM:NVP)	1:4:5
Total monomer concentration	30%
Cross-linker concentration	0.4%
Emulsifier concentration	10%
Initiator concentration	0.08%
Reaction temperature	50 °C
Reaction time	12 h
Rotation speed	460 rotations per minute (RPM)

**Table 3:** The synthesis conditions of AMN other than monomer ratio.

Synthesis Conditions	Various Parameters
HLB	5.1
Oil–water ratio	1:1
Total monomer concentration	30%
Cross-linker concentration	0.4%
Emulsifier concentration	10%
Initiator concentration	0.08%
Reaction temperature	50 °C
Reaction time	12 h
Rotation speed	460 rotations per minute (RPM)

**Table 4:** The synthesis conditions of AMN other than total monomer concentration.

Synthesis Conditions	Various Parameters
HLB	5.1
Oil–water ratio	1:1
Monomer ratio (AMPS:AM:NVP)	1:4:5
Cross-linker concentration	0.4%
Emulsifier concentration	10%
Initiator concentration	0.08%
Reaction temperature	50 °C
Reaction time	12 h
Rotation speed	460 rotations per minute (RPM)

**Table 5:** The synthesis conditions of AMN other than cross-linker concentration.

Synthesis Conditions	Various Parameters
HLB	5.1
Oil–water ratio	1:1
Monomer ratio (AMPS:AM:NVP)	1:4:5
Total monomer concentration	30%
Emulsifier concentration	10%
Initiator concentration	0.08%
Reaction temperature	50 °C
Reaction time	12 h
Rotation speed	460 RPM

## Data Availability

Not applicable.
